# Comparison of the Effects of Resveratrol and Its Derivatives on the Radiation Response of MCF-7 Breast Cancer Cells

**DOI:** 10.3390/ijms22179511

**Published:** 2021-09-01

**Authors:** Dominika Komorowska, Agnieszka Gajewska, Paweł Hikisz, Grzegorz Bartosz, Aleksandra Rodacka

**Affiliations:** 1 Department of Molecular Biophysics, Faculty of Biology and Environmental Protection, University of Lodz, 141/143 Pomorska St., 90-236 Lodz, Poland; dominika.komorowska@edu.uni.lodz.pl (D.K.); agatazator@wp.pl (A.G.); pawel.hikisz@biol.uni.lodz.pl (P.H.); 2 Department of Bioenergetics, Food Analysis and Microbiology, Institute of Food Technology and Nutrition, College of Natural Sciences, Rzeszow University, 4 Zelwerowicza St., 35-601 Rzeszow, Poland; grzegorz.bartosz@gmail.com

**Keywords:** natural polyphenols, stilbene derivative, ionising radiation, MCF-7 cells, breast cancer, radiotherapy

## Abstract

Radiotherapy is among the most important methods for breast cancer treatment. However, this method’s effectiveness is limited by radioresistance. The aim of this study was to investigate whether the stilbene derivatives piceid, resveratrol, and piceatannol have a radiosensitising effect on breast cancer cells (MCF-7). The conducted research enabled us to determine which of the tested compounds has the greatest potential in sensitising cells to ionising radiation (IR). Among the stilbene derivatives, resveratrol significantly increased the effect of IR. Resveratrol and IR used in combination had a higher cytotoxic effect on MCF-7 cells than using piceatannol, piceid, or radiation alone. This was due to a significant decrease in the activity of antioxidant enzymes, which resulted in the accumulation of formed reactive oxygen species (ROS). The effect of resveratrol and IR enhanced the expression of apoptotic genes, such as *Bax*, *p53*, and *caspase 8*, leading to apoptosis.

## 1. Introduction

Cancer is the second most common cause of death in the world, after cardiovascular diseases. Each year, there are 18,100,000 new cancer cases in the world. Breast cancer is the most commonly diagnosed type of cancer and the leading cause of cancer-related deaths in women, with approximately 1,500,000 new cases and 400,000 deaths each year [1]. Currently, the basic methods of treatment of breast cancer include surgical tumour removal, chemotherapy, endocrine therapy and radiotherapy. Half of metastatic breast cancers respond to endocrine therapy. Postoperative adjuvant hormone therapy reduces disease relapse by approximately 50% [2]. Radiotherapy is aimed at destroying cancerous changes using ionising radiation (IR); however, in some cases, solid epithelial breast tumours are observed to be resistant to the apoptosis that is induced by IR. Despite the fact that radiotherapy has huge potency, it also poses risks for patients since normal cells are also sensitive to radiation-induced damage. Therefore, a lot of studies are aimed at developing new drugs that can protect normal cells from radiation-induced apoptosis and increase the toxic effect of IR in cancer cells [3].

In recent years, compounds of plant origin—called phytochemicals—have been the subject of many studies and have been identified as potential radiosensitisers that enhance the sensitivity of cancer cells to IR [4,5]. Many of them are also considered antitumour drugs [6,7].

The most popular polyphenol and powerful antioxidant is resveratrol (3,4,5-*trans*-trihydroxystilbene, abbreviation R) [8,9,10]. The highest concentrations of this compound have been observed in grapes, berries, and peanuts.

**Figure 1:**
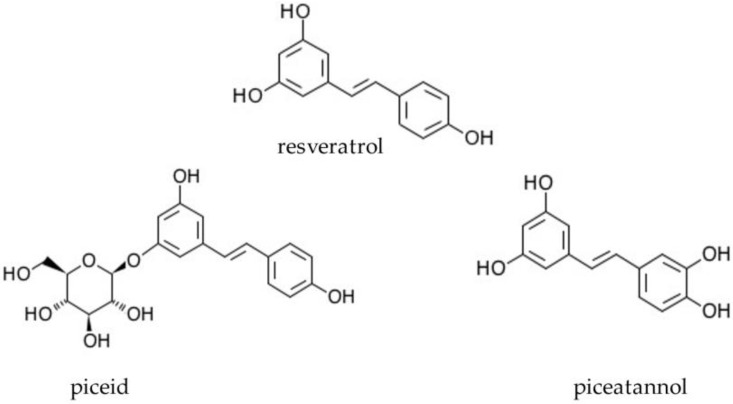
Chemical structure of resveratrol and its derivatives, piceatannol and piceid.

Naturally occurring analogues of resveratrol are piceatannol (3-hydroxyresveratol, abbreviation ROH) and piceid, also known as polydatin (trans-resveratrol substituted at position 3 by a beta-d-glucosyl residue, abbreviation RG) (Figure 1). Piceid is a natural precursor and the glycoside form of resveratrol. In addition, it is the most abundant form of resveratrol in nature [11]. A number of studies have suggested that piceid may have a bioactivity that is similar to resveratrol [12,13]. Piceatannol is a resveratrol metabolite found in red wine, white tea, passion fruit, and Japanese knotweed. Studies have shown that these compounds possess many different biological properties, such as anti-inflammatory, immunoregulatory, antioxidative, and antitumour activities. The antitumour activity of stilbene derivatives is mediated mainly by the signalling pathways associated with gene expression and the induction of cell apoptosis [14,15].

In this study, we aimed to investigate whether resveratrol and its derivatives, piceatannol and piceid, could sensitise breast cancer cells (MCF-7) to ionising irradiation. In all experiments, cells were pretreated with the stilbene derivative for 3 h (5 or 25 µM) and/or with IR (doses of 2 or 6 Gy), and then the following series of assays were performed: cell viability assays (MTT assay), FITC Annexin V staining (for determination of the percentage of cells that were actively undergoing apoptosis), apoptotic gene expression assays (*p53*, *Bax*, *Bcl-2*, *caspase 3*, and *caspase 8*), Western blotting analyses and antioxidant enzyme activity assays (catalase [CAT], superoxide dismutase [SOD], and glutathione peroxidase [GPx] activity).

## 2. Results

### 2.1. Cell Viability

**Figure 2:**
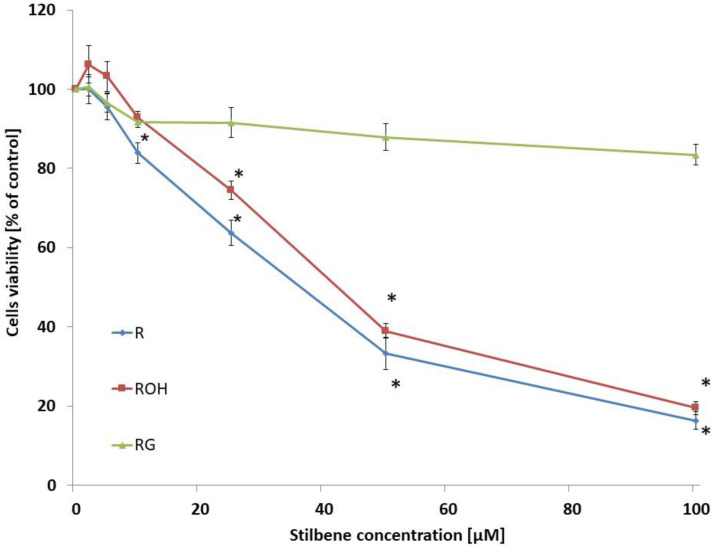
Effect of resveratrol (R), piceatannol (ROH), and piceid (RG) on MCF-7 viability. Cell viability was estimated with the MTT test and analysed after 48 h of treatment of the MCF-7 cells with stilbene at concentrations of 0–100 µM. The presented data are the average of five independent experiments, shown as the mean ± SD (* *p* < 0.05, Student’s *t*-test).

In the first stage of our study, we investigated the cytotoxic effect of the studied compounds on MCF-7 cells incubated for 48 h. These compounds were used at concentrations of 2.5–100 µM. The control consisted of cells that were not treated with the compounds. Of the tested compounds, resveratrol was the most cytotoxic. Piceatannol showed a slightly lower cytotoxicity, whereas piceid did not significantly affect metabolic viability. Incubation of the cells with resveratrol for 48 h resulted in a statistically significant decrease in viability above a concentration of 25 μM. Piceatannol caused a statistically significant decrease in viability above a concentration of 50 μM (Figure 2). Piceid did not alter viability in the concentration range studied. For further studies, compounds were used at concentrations of 5 or 25 µM.

**Figure 3:**
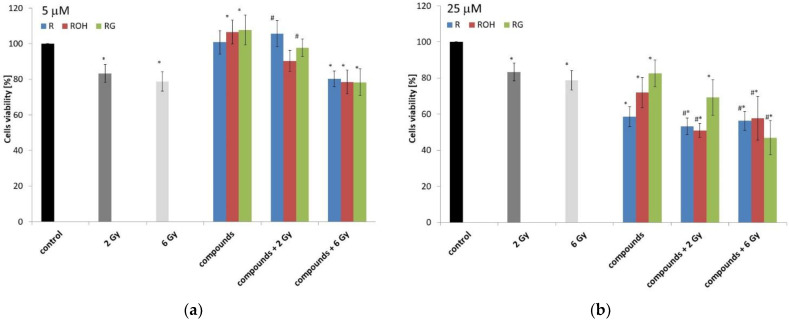
The MCF-7 cell viability under the effect of 5 (**a**) or 25 μM (**b**) of resveratrol (R), piceatannol (ROH), and piceid (RG) alone and in combination with ionising radiation (IR) (dose of 2 or 6 Gy) was determined using an MTT test after 48 h of incubation. All results are presented as the mean ± SD of three independent repetitions. Treatments were compared by a two-way analysis of variance (ANOVA) followed by Tukey’s post hoc test (*p* < 0.05). The mean difference (*) was compared with the control and (#) with IR.

Next, we determined the cytotoxicity of the tested compounds in combination with the action of IR in doses of 2 or 6 Gy. IR alone (at both doses used) caused a decrease in the metabolic viability of MCF-7 cells after 48 h of incubation (Figure 3a,b). There was no difference in viability depending on the dose. At doses of both 2 and 6 Gy, the decrease in viability was about 20% (Figure 3).

Forty-eight hours after irradiation, we observed an increase in the viability of cells incubated with the tested compounds (concentration: 5 µM) compared to the control. Similarly, we observed an increase in cell viability under combination therapy (preincubation with stilbenes at a concentration of 5 µM and treated with an IR dose of 2 Gy) compared to cells treated with IR alone (Figure 3a). The use of a higher dose of 6 Gy resulted in a decrease in viability, similar to that observed for cells exposed to radiation alone. Metabolic viability significantly decreased in cells preincubated with polyphenols at a concentration of 25 µM and treated with IR at doses of 2 and 6 Gy.

### 2.2. Apoptosis

**Figure 4:**
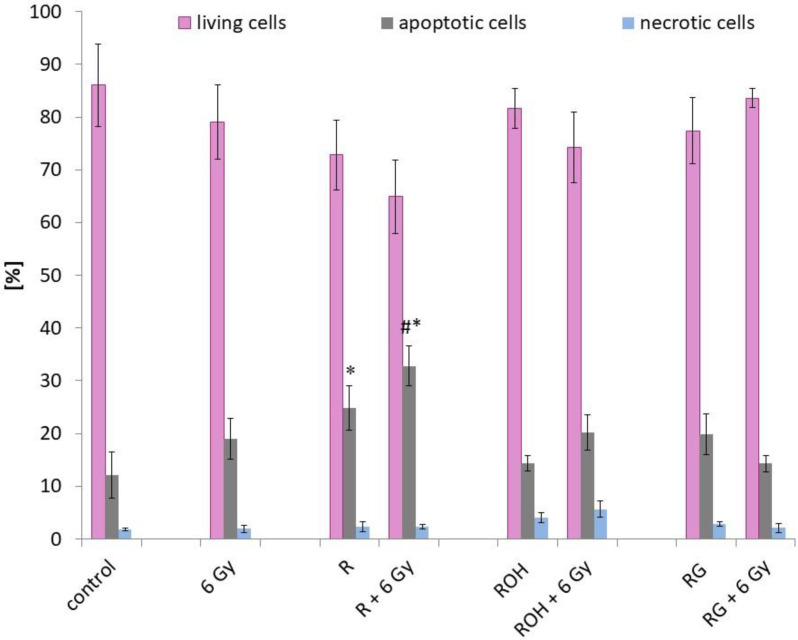
Apoptosis in MCF-7 cells treated with 25 μM of resveratrol (R), piceatannol (ROH), and piceid (RG) alone and in combination with IR (dose of 6 Gy). Annexin V and propidium iodide staining in MCF-7 cells incubated with stilbene and/or IR for 24 h. Data are shown as the mean ± SD of three independent repetitions. Treatments were compared by a two-way analysis of variance (ANOVA) followed by Tukey’s post hoc test (*p* < 0.05). The mean difference (*) was compared with the control and (#) with IR.

Apoptotic and necrotic changes were determined by flow cytometry using an Annexin V Apoptosis Detection Kit. Figure 4 shows the fraction of live, apoptotic, and dead cells. The study was performed on cells that were preincubated with the tested compounds at a concentration of 25 μM and then irradiated with a dose of 6 Gy and incubated for 24 h.

Among the tested compounds, only resveratrol significantly induced apoptosis in MCF-7 cells (about 20%) compared to the control (cells not treated with the compounds and not exposed to radiation). In cells preincubated with resveratrol and then exposed to IR, apoptosis was further induced. Apoptosis increased to about 33% (an increase of approximately 10% compared to cells treated only with resveratrol, and an increase of about 15% compared to cells treated with radiation alone). In samples preincubated with piceatannol or piceid and then subjected to radiation, no significant increase in the percentage of apoptotic cells was observed compared to the samples subjected to only radiation.

### 2.3. Apoptotic Gene Expression

**Figure 5:**
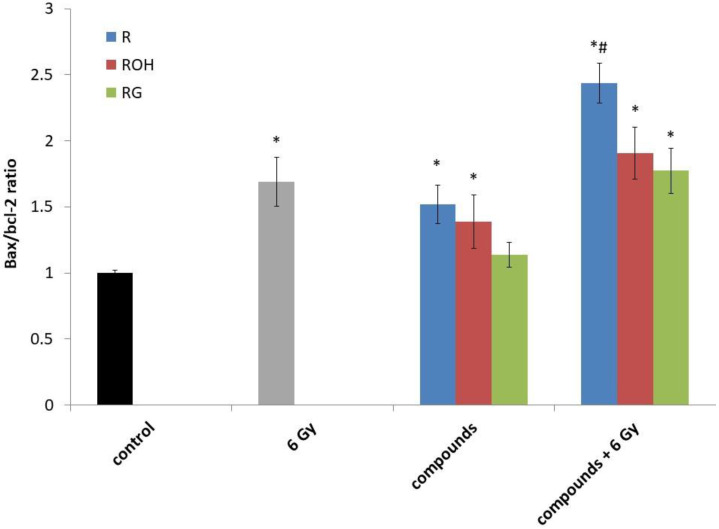
The *Bax/Bcl-2* ratio in the MCF-7 cells treated with 25 μM of resveratrol (R), piceatannol (ROH), and piceid (RG) alone and in combination with IR (dose of 6 Gy). MCF-7 cells were incubated with stilbene and/or IR for 24 h. Data are the results of three independent repetitions. Treatments were compared by a two-way analysis of variance (ANOVA) followed by Tukey’s post hoc test (*p* < 0.05). The mean difference (*) was compared with the control and (#) with IR.

In MCF-7 cells, we determined the expression of the following five genes related to the cell death process: *Bax*, *Bcl-2*, *caspase 3*, *caspase 8*, and *p53* (Table 1 and Figure 5). Gene expression was analysed by real-time PCR. Cells were incubated with the compounds at 25 μM and irradiated with a dose of 6 Gy. Changes in the expression of the selected genes were examined after 24 h of incubation.

A statistically significant overexpression of *p53* was observed under all tested models—i.e., for cells exposed to radiation alone, cells treated with stilbene derivatives alone, and cells treated with the compounds and radiation. The highest expression was induced by resveratrol alone and resveratrol used in combination with radiation.

Expression of the proapoptotic *Bax* gene and the antiapoptotic *Bcl-2* gene followed the expression ratio of *Bax/Bcl-2* (Figure 5). We observed an increase in the *Bax/Bcl-2* ratio in all systems tested. The highest increase in *Bax* expression relative to *Bcl-2* was observed for resveratrol used in combination with IR. The highest level of apoptosis was also observed in this combination (Figure 4). To determine the apoptotic pathway induced by stilbene derivatives alone or in combination with IR, the expression of *caspase 3* and *caspase 8* was also measured.

In general, the increase in *caspase 3* expression in MCF-7 cells was relatively low. Among the tested derivatives, only resveratrol increased *caspase 3* expression. All compounds in combination with radiation increased *caspase 3* expression at the level of expression observed for only irradiated cells. For *caspase 8*, an increase in expression was observed in all tested systems—i.e., for cells exposed to radiation alone, cells treated with stilbene derivatives alone, and cells treated with the compounds and radiation.

### 2.4. Western Blot Analysis

**Figure 6:**
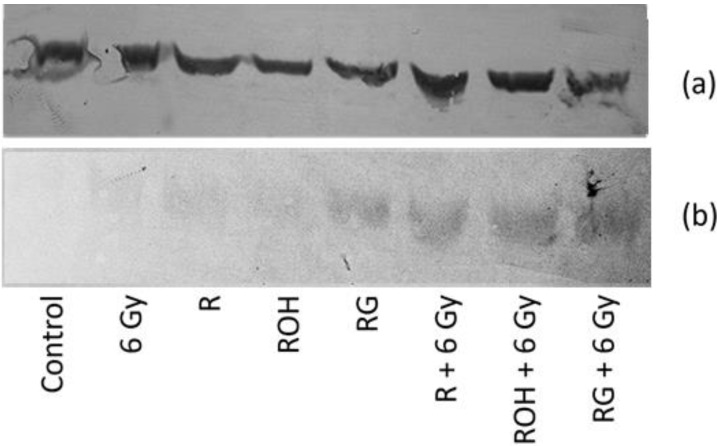
Protein levels of β-actin (**a**) and caspase 8 (**b**) in MCF-7 cells treated with 25 μM of resveratrol (R), piceatannol (ROH), and piceid (RG) alone and in combination with IR (dose of 6 Gy). MCF-7 cells were incubated with stilbene and/or IR for 24 h. The lysates were resolved by SDS-PAGE and analysed via the Western blot technique; primary mouse antibodies β-actin (1:1000 dilution) and caspase 8 (1:500).

Western blot analysis confirmed the increase in caspase 8 expression in MCF-7 cells treated with the studied stilbene and irradiated together (Figure 6). We did not observe any caspase 3 expression in MCF-7 cells, which is consistent with the data in the literature [16].

### 2.5. Antioxidant Enzyme Activities

**Figure 7:**
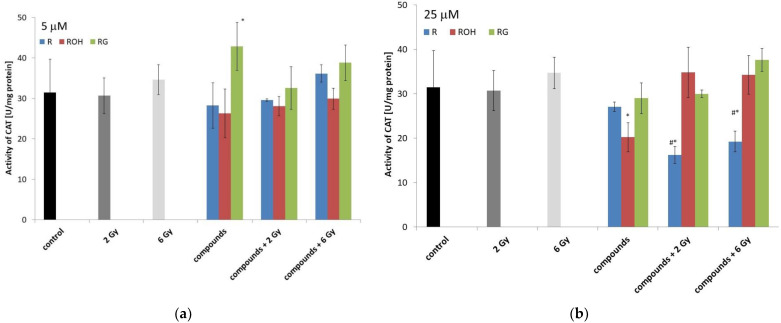
Effect of resveratrol (R), piceatannol (ROH), piceid (RG), ionising radiation, and their combinations on the catalase (CAT) activity of MCF-7 cells. CAT activity was measured after incubation of the cells for 48 h. Cells were treated with stilbene derivatives at concentrations of 5 μM (**a**) and 25 μM (**b**). The presented data are the averages of three independent experiments, shown as the mean ± SD. Treatments were compared by a two-way analysis of variance (ANOVA) followed by Tukey’s post hoc test (*p* < 0.05). The mean difference (*) was compared with the control and (#) with IR.

**Figure 8:**
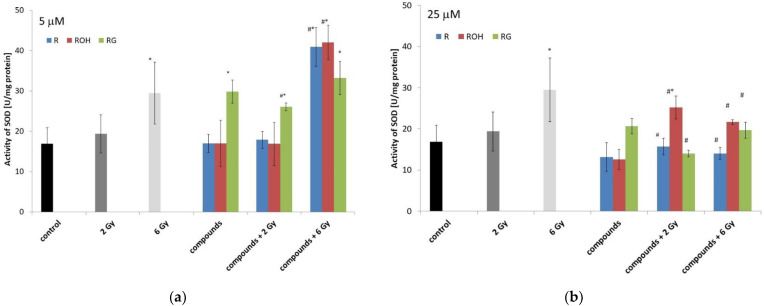
Effect of resveratrol (R), piceatannol (ROH), piceid (RG), ionising radiation, and their combinations on the superoxide dismutase (SOD) activity of MCF-7 cells. SOD activity was analysed after incubation of the cells for 48 h. Cells were treated with stilbene derivatives at concentrations of 5 μM (**a**) and 25 μM (**b**). All data are the average of three independent experiments, shown as the mean ± SD (two-way analysis of variance (ANOVA) followed by Tukey’s post hoc test, *p* < 0.05). The mean difference (*) was compared with the control and (#) with IR.

**Figure 9:**
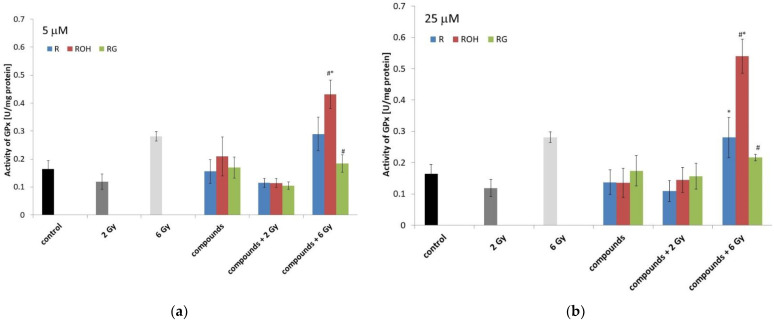
Effect of resveratrol (R), piceatannol (ROH), piceid (RG), ionising radiation, and their combinations on the glutathione peroxidase (GPx) activity of MCF-7 cells. GPx activity was measured after incubation of the cells for 48 h. Cells were treated with stilbene derivatives at concentrations of 5 μM (**a**) and 25 μM (**b**). The presented data are the average of three independent experiments, shown as the mean ± SD (two-way analysis of variance (ANOVA) followed by Tukey’s post hoc test, *p* < 0.05). The mean difference (*) was compared with the control and (#) with IR.

In this study, the enzymatic activity of three antioxidant enzymes, CAT, SOD, and GPx was determined (Figure 7, Figure 8 and Figure 9).

#### 2.5.1. Catalase

Under the influence of radiation alone, after 48 h of incubation, we did not observe any significant changes in CAT activity (Figure 7).

Incubation of the cells for forty-eight hours with low concentrations of piceid alone significantly increased CAT activity by approximately 12% compared to the control. At a concentration of 5 µM, resveratrol and piceatannol did not change the enzyme activity (Figure 7a).

At a higher concentration of the tested compounds (25 µM), a significant decrease in CAT activity was observed for cells preincubated with resveratrol and then irradiated with doses of 2 and 6 Gy.

#### 2.5.2. Superoxide Dismutase

With an increase in dose radiation, we observed an increase in enzyme activity compared to the control. Resveratrol and piceatannol alone at both applied concentrations did not statistically change the enzyme activity. Piceid alone at a concentration of 5 μM produced a statistically significant increase in SOD activity.

We observed a statistically significant decrease in SOD activity in cells treated with resveratrol or piceid at a concentration of 25 μM and then irradiated, compared to that in cells treated only with ionising radiation. A significant increase in SOD activity was observed in cells preincubated with piceid at a concentration of 5 μM and then irradiated with a dose of 2 Gy. Application of the 6 Gy dose did not significantly affect the activity of the enzymes in cells preincubated with piceid. However, we observed an increase in enzyme activity in the cells preincubated with resveratrol or piceatannol. In most cases, the treatment of cells with 25 μM of polyphenol followed by irradiation significantly reduced the activity of SOD.

#### 2.5.3. Glutathione Peroxidases

The effect of radiation and polyphenols alone on MCF-7 cells did not significantly affect the activity of GPx (Figure 9). A significant increase in enzyme activity was also observed in cells preincubated with piceatannol (at both concentrations used) and irradiated with a dose of 6 Gy.

## 3. Discussion

Breast cancer is the most frequently detected malignancy in women and a major cause of cancer death among women worldwide [17]. Radiotherapy is among the most important methods of cancer treatment. Radiotherapy is not only considered a primary therapy but is also used alongside chemotherapy, hormone therapy, and surgery [18,19]. Nonetheless, radioresistance and side effects are limiting factors of this method for breast cancer treatment. Therefore, studying substances that can enhance the radiation effect and protect normal cells is highly relevant. Many studies have demonstrated that several bioactive food components, such as polyphenols (curcumin, genistein, and quercetin), can increase the radiosensitivity of tumour cells [20,21]. The latest studies have shown that resveratrol intensifies the radiosensitivity of breast cancer cells (MCF-7), prostate cancer cells (PC3), and nasopharyngeal carcinoma cells (CNE-1) [22,23,24,25].

The aim of this study was to investigate whether stilbene derivatives, i.e., piceid, resveratrol, and piceatannol, can affect the radiosensitising effect on breast cancer cells (MCF-7). In addition, the conducted research allowed us to determine which of the tested compounds had the greatest potential in sensitising cells to IR.

The concentrations of the tested compounds used in the present study were 5 and 25 μM. The greatest cytotoxic effect on MCF-7 cells, assessed using an MTT test, was observed at a concentration of 25 µM. The most cytotoxic compound was resveratrol (yielding a decrease in metabolic viability of about 41%), and the least cytotoxic was piceid (yielding a decrease of about 17%). The use of resveratrol and the other tested compounds at a concentration of 5 µM did not significantly affect the metabolic viability, apoptosis, or activity of antioxidant enzymes in the cells treated with the compound alone or in combination with IR. In many cases, we observed a significant stimulation of MCF-7 cells (increased viability and activity of antioxidant enzymes). A concentration-dependent impact of resveratrol, piceatannol, and another small natural compounds on cancer cells was previously observed by other authors [24,26,27,28]. Low concentrations promoted the viability and proliferation of a variety of cancer cell lines (i.e, a procarcinogenic effect), while higher concentrations had an anticarcinogenic effect [24,26,29].

Subsequently, we determined which of the studied compounds increased the cytotoxic effect of ionising radiation on the cells. It was shown that piceid at a concentration of 25 µM together with IR (6 Gy) can reduce metabolic viability to the greatest extent (a reduction of around 14% compared to the additive effect of piceid and IR). In the remaining cases, it was shown that the tested compounds (25 µM) in combination with radiation reduced the metabolic viability of MCF-7 cells to a lesser extent compared to the sum of the effects of the studied stilbenes and IR. Resveratrol in combination with radiation decreased metabolic viability to the same extent as the compound alone, without an additive effect. Rai et al. showed that radiation induces mitochondrial biogenesis and hyperactivation, leading to increased metabolic viability and MTT reduction. The extent of the radiation-induced reduction in cell numbers was found to be larger than the decrease in MTT reduction in many cell lines tested. The tetrazolium salts used in the MTT assays to measure the mitochondrial metabolic rate did not correlate with actual percentage of terminated cells [30].

We then estimated the extent to which the tested compounds, in combination with radiation, induced cell death. This evaluation was performed using the fluorimetric method with a high-sensitivity Annexin V-FITC Apoptosis Kit. We also attempted to assess the rate of apoptosis and necrosis 48 h after irradiation was performed (i.e., incubation time, after which the viability and activities of the antioxidant enzymes were assessed). However, the obtained results were unreliable. Apoptosis, which involves the activation of proteins from the BCl-2 family and depolarisation of the mitochondria, is a relatively rapid process that occurs within a few hours of the applied stimulus [31,32]. Finally, the rate of apoptosis was measured 24 h after IR exposure. Our study showed that among the selected compounds, only resveratrol statistically significantly induced apoptosis in cells treated with resveratrol alone, as well as in cells exposed to resveratrol and IR. For the remaining stilbene derivatives (piceatannol and piceid) used alone or in combination with radiation, we did not observe a statistically significant increase in the level of apoptosis compared to the control cells or the cells that were irritated only. Our results indicate that among the studied stilbene derivatives, resveratrol has the greatest ability to strengthen the effect of IR in the MCF-7 cell line. Recently published studies by Amini et al. also showed that resveratrol potentiated the effects of radiation on MCF-7 cells [33].

Research showed that the balance between proapoptotic and antiapoptotic proteins, mitochondrial dysfunction, caspase activities, and level of reactive oxygen species (ROS) are the most important factors involved in programmed cell death in tumour cells [32,34,35]. Higher levels of ROS are observed in cancer cells compared to normal cells. Despite this, cancer cells maintain their redox balance due to their high antioxidant capacity [36]. A high level of oxidative stress is considered a novel target for anticancer therapy. This can be achieved by increasing exogenous ROS and/or inhibiting the antioxidant system [37,38].

In this work, we demonstrated that the level of apoptotic changes in MCF-7 cells treated with the studied stilbenes and IR is related to, among other factors, changes in the activities of antioxidant enzymes (catalase and superoxide dismutase). Resveratrol in combination with radiation significantly reduced CAT activity and, to a lesser extent, SOD activity. The decreased activity of these enzymes led to the accumulation of ROS in the cells exposed to radiation [37,39].

Excessive amounts of ROS may act as cellular toxicants, which can lead to cancer-cell growth arrest, apoptosis, and necrosis. It is speculated that malignant cells under increased levels of oxidative stress are more vulnerable to further ROS attacks [40]. The radiotherapy strategy is based on IR, which increases ROS generation and induces apoptotic damage in cancer cells [41].

It was observed that the tested compounds alone did not significantly change the activity of the antioxidant enzymes in MCF-7 cells. The only exception was piceid, which at a lower concentration, significantly stimulated the activity of catalase and superoxide dismutase. Our results confirm earlier reports showing that piceid enhances the endogenous antioxidant defence system, especially increasing superoxide dismutase and catalase activities [42,43,44,45]. In this way, piceid effectively protects cells experiencing the induced overproduction of free radicals [46]. Based on this study, we determined that the antioxidant capacity of piceid is ten times stronger than that of resveratrol [44]. Additionally, in a study by Su et al. (2013), piceid exhibited higher scavenging activity than resveratrol against hydroxyl radicals [47].

In cells treated with piceatannol or piceid, the IR activity of the studied enzymes did not change significantly compared to the control. In some cases, we observed that the combination treatment of MCF-7 cells significantly enhanced the activity of the studied enzymes. For example, piceatannol significantly increased GPx activity under conditions of enhanced oxidative stress. The stimulating effect of piceatannol on the activity of GPx was also observed in an earlier work on neuroblastoma cells [48]. The effective action of antioxidant enzymes in cells protects those cells against the ROS generated by radiation.

In the irradiated cells themselves, we observed a dose-dependent increase in the activity of the studied enzymes, especially for SOD and GPx. The increased activities of antioxidant enzymes after ionising irradiation are well known. Our research confirmed that mammalian cells can generate an SOS-like response, similar to that previously described by other authors in prokaryotic cells [49,50]. The enhanced antioxidant potential of tumour cells after irradiation is connected with radioresistance [51,52].

One property of resveratrol is its inhibition of cell survival signalling, which may directly stimulate the signalling cascade of the apoptotic pathway or block the antiapoptotic mechanisms in this situation [53]. Resveratrol can sensitise cancer cells and enhance antitumour activities when it is used in combination with another therapy, such as chemotherapy or radiotherapy [15,22,23,24,25]. In our study, we showed that resveratrol alone and, to a greater extent, resveratrol in combination with IR enhance *p53* gene expression.

It has been well studied that in unstressed cells p53 protein levels are very low because it is targeted for proteasomal degradation. This protein is activated in response to many stress stimuli, including reactive oxygen species [54,55]. The p53 protein stimulates a wide network of signals that act through two major apoptotic pathways: extrinsic pathways and intrinsic pathways [56]. The extrinsic, death receptor pathway induces the activation of a caspase cascade, and the intrinsic, mitochondrial pathway shifts the balance in the Bcl-2 family of intracellular proteins towards the pro-apoptotic members [56]. Pro-apoptotic members of the Bcl-2 family (Bax, Bak etc.) induce the release of cytochrome c and cause mitochondrial dysfunction. In contrast, antiapoptotic members such as Bcl-2 work as protectors of the outer membrane, and preserve its integrity by suppressing the release of cytochrome c [57]. The balance of anti- (*Bcl-2*) and proapoptotic (*Bax*) genes can determine the fate of cancer cells. We next examined the effects of the stilbenes alone and in combination with IR on the gene expression of *Bax* and *Bcl-2*. The results of our analysis show that the *Bax:Bcl-2* gene expression ratio was significantly higher after treatment with a combination of resveratrol and ionising radiation compared to that in the groups treated with resveratrol or ionising radiation alone (Figure 5). Our results suggest that resveratrol and IR in combination greatly intensify apoptotic signal transmission. A lower rate of apoptosis in cells treated with resveratrol analogues, i.e., piceatannol or piceid (also in combination with IR), correlated to a lower expression of *p53* and a smaller increase in the *Bax/Bcl-2* ratio (Figure 5 and Table 1). Similar effects of resveratrol on MCF-7 cells were observed by, among others, Mirzapur et al. [35].

Previous studies showed that resveratrol in MCF-7 cells cannot affect the activities of caspase 3 and caspase 8 in the apoptosis process [58,59]. In turn, studies by Mirzapur showed that resveratrol increases the expression of both caspases 3 and 8 in MCF-7 cells [35]. In addition, some studies have shown that MCF-7 breast cells do not express caspase 3 [16,60]. Taking into account the discrepancies in the literature data in this study, we investigated the expression of two caspases, initiator caspase 8 and executive caspase 3. In our research, we observed very low expression of the caspase 3 gene only in irradiated cells in the absence or presence of polyphenols. As expected, the results of our Western blot analyses directly demonstrated the absence of caspase 3 in MCF-7 cells. The very low gene expression of *caspase 3* in our research may be partially explained by the use of inappropriate primers that could amplify non-functional caspase 3 mRNA [61].

We and others have shown that the absence of caspase 3 did not prevent death in MCF-7 cells [62]. Wang et al. demonstrated that MCF-7 cells underwent cell death, utilising an atypical apoptosis pathway, at an insignificant, slower rate, compared to that in caspase 3-expressing MCF-7 cells, and in A431 cells, which underwent typical intracellular apoptosis [63]. Kagawa et al. evaluated the role of caspase 3 in Bax-induced apoptosis. In the research they used caspase 3-deficient MCF7 cells and clones stably transfected with the caspase 3 gene (MCF7/Casp3). The results revealed that caspase 3 is not required for Bax-mediated cell death itself. They also demonstrated that in MCF-7 cells caspase 6 can be activated even in the absence of caspase 3 [62]. Our study confirmed that a lack of caspase 3 did not impact Bax-induced apoptosis in MCF-7 cells.

Caspase 3 is crucial for apoptosis induction, as this enzyme is not only activated downstream of both the extrinsic and intrinsic death pathway but is also responsible for DNA fragmentation. Studies by other authors showed that in MCF-7 cells lacking caspase 3, apoptosis proceeds via the sequential activation of caspases 9, 7, and 6 [61,64,65]. On the basis of the temporal sequence of caspase activation in neocarzinostatin-treated MCF-7 cells, Liang et al. proposed apoptosis cascade in these cells. According to the obtained data, decreased Bcl-2 and increased Bax levels induce the release of cytochrome c from the mitochondria. Cytochrome c activates caspase 9, which in turn activates caspase 7. Activated caspase 7 activates caspase 6, which induces apoptosis in MCF-7 cells, presumably through cleavage of nuclear lamins [61].

Our study also showed that all tested stilbenes alone or in combination with IR increase the expression of caspase 8, which is a characteristic of the receptor pathway for apoptosis activation. A Western blot analysis of caspase 8 was also performed. Despite the poor quality of the blot, we decided to include the results to confirm the increase in caspase 8 expression, especially in systems where cells were exposed to both stilbene and radiation. We were unable to densitometrically assess and compare the levels of proteins in the tested systems.

The role of caspase 8 in resveratrol-induced apoptosis in MCF-7 has been confirmed by other authors [25,35]. Caspase 8 activation can induce death via direct cleavage of caspase 7, or can cleave the Bcl-2 family protein BID that can activate intrinsic apoptosis [66,67].

Our results suggest that the most likely mechanism of resveratrol- and IR-induced apoptosis in MCF-7 cells is ROS generation with the involvement of either intrinsic or extrinsic apoptotic pathways.

**Figure 10:**
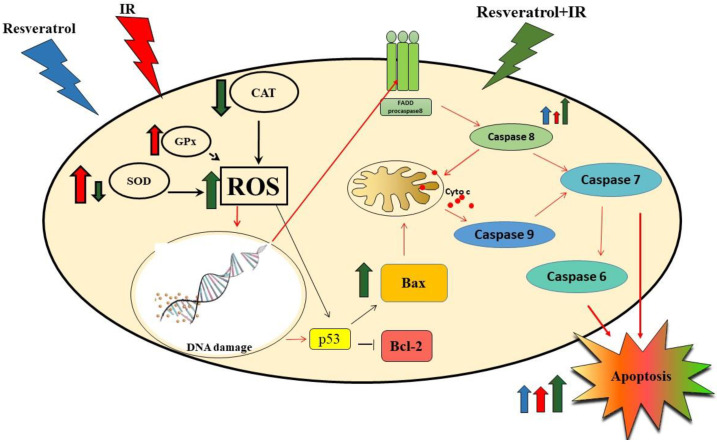
This image shows only the pathways of the resveratrol-induced radiosensitivity mechanism in MCF-7 cells that were investigated in this work (black arrows) or discussed on the basis of literature data (red arrows). Resveratrol potentiated the radiosensitivity in MCF-7 cells by decreasing antioxidant enzyme activity (SOD and CAT). Inhibiting SOD and CAT activity would result in increased ROS, thereby causing damage to cellular macromolecules. DNA damage leads to activation of the p53 protein. The p53 protein mainly acts as a transcription factor that induces and/or inhibits the activation of many genes associated with the induction of apoptosis. In the intrinsic pathway of apoptosis, p53 may directly activate the proapoptotic Bax protein or inhibit the antiapoptotic bcl-2 protein. The Bax protein causes the opening of mitochondrial channels and the release of cytochrome c. Cyt c, along with other proapoptotic agents, activates caspase 9, which affects the activation of executive caspases. DNA damage can also induce the extrinsic pathway of apoptosis associated with the activation of surface death receptors (FAS). The changes in the conformation of these receptors after attaching to the FADD protein lead to the activation of caspase 8, which can induce death via direct cleavage of caspase 7, or can activate intrinsic apoptosis. Thus, resveratrol enhances the process of apoptosis in MCF-7 cells that are exposed to IR.

In conclusion, among the selected stilbene derivatives, resveratrol most significantly increased the effect of IR. Resveratrol and IR used in combination had a higher cytotoxic effect on MCF-7 cells than using piceatannol, piceid, or radiation alone. This effect was, among other factors, due to a significant decrease in the activity of antioxidant enzymes, resulting in the accumulation of formed ROS. The effects of resveratrol and IR were found to enhance the expression of apoptotic genes, such as *Bax*, *p53*, and *caspase 8*, leading to apoptosis (Figure 10).

The presented basic research helps to better understand the mechanism of action of resveratrol in breast cancer cells under the conditions of increased oxidative stress. However, these results cannot be directly translated into physiological conditions. The biological effect of resveratrol in vivo appears to be strongly limited by its low bioavailability, which is a barrier for the development of therapeutic applications. As clinical trial demonstrated, no free RSV was observed in both malignant and normal breast tissues in breast cancer patients who consumed a dietary blend of polyphenols, including RSV [68]. Orally administered resveratrol, like other polyphenols, has poor bioavailability and is converted into a wide variety of metabolites. Growing evidence highlights that mainly glucuronide and sulfate conjugates metabolites of resveratrol are the molecules that could reach systemic human tissues [69,70]. Ávila-Gálvez et al. showed, that in normal and malignant mammary tissues from breast cancer patients major metabolites of resveratrol are resveratrol-3-O-sulfate and dihydro resveratrol-3-O-glucuronide. Among these, the percentage of sulphated was slightly lower in normal tissues (31%) than in tumour (42%). In both tissues, resveratrol metabolites showed levels in the range of the low nM [71]. In analogous study performed on rats, the concentration of resveratrol metabolites was shown to be in the low µM range. In this case higher proportion of RSV sulphates than glucuronides (opposite to what is observed in humans) was observed [72]. Studies have shown that conjugated metabolites are less bioactive than their free forms [68]. Despite this, Giménez-Bastida et al. demonstrated for the first time that physiologically relevant RSV metabolites can promote a moderate cellular senescence induction in breast cancer cells. They also demonstrated that these metabolites are not deconjugated to release free RSV but enter the cells through ABC transporters [68].

Studies that show that metabolites of RSV are less bioactive than their free forms confirm that further studies on the effects of free resveratrol in biological systems are warranted. The well-documented biological effectiveness of resveratrol is the basis for further explore methods to optimize bioavailability in humans. Many of the strategies to increase bioavailability of resveratrol are described in detail by Amri et al. [73] and Smoliga et al. [74]. High hopes are placed on nanotechnology. Modern and intelligent nanocarriers are able to combine protection, controlled release and targeting functionalities.

## 4. Materials and Methods

### 4.1. Cell Line

The MCF-7 (ATCC, Manassas, VA, USA) human breast carcinoma cell line was used. The cells have the ability to process estradiol via cytoplasmic estrogen receptors. MCF-7 growth was inhibited by tumour necrosis factor alpha (TNFα). MCF-7 were grown in Dulbecco’s modified Eagle’s medium (DMEM) supplemented with 10% foetal bovine serum and antibiotics (10 U/mL penicillin and 50 μg/mL streptomycin). The cells were incubated in 5% CO_2_/95% air at 37 °C. After reaching 80–90% confluence, cells were carefully removed with trypsin/EDTA and washed with fresh phosphate-buffered saline (PBS). Cell viability was determined using the trypan blue assay.

### 4.2. Chemicals and Reagents

Cell culture media, PBS, foetal bovine serum, antibiotics, and MTT were obtained from Sigma–Aldrich (St. Louis, MO, USA). The Annexin V-FITC Apoptosis Detection Kit was obtained from Molecular Probes. The RNA isolation Kit EXTRACTME reagent was obtained from BLIRT. The RevertAid First Strand cDNA Synthesis Kit was obtained from Applied Thermo Scientific. 5x HOT FIREPol^®^ EvaGreen^®^ qPCR Supermix was from Solis Biodyne. Oligonucleotide, p53, Bax, Bcl-2, caspase 3, caspase 8, and hypoxanthine phosphoribosyltransferase (HPRT) were purchased from Genomed. Antibodies specific to caspases 3, 8 and β-actin were obtained from Santa Cruz Biotechnology. Secondary antibodies were purchased from Sigma–Aldrich.

In the experiments, three chemical compounds from the stilbenes group were used: resveratrol (3,4′,5-*trans*-trihydroxystilbene, R), piceatannol (3,3′,4,5′-*trans*-trihydroxystilbene, ROH)—a naturally occurring hydroxylated analogue of resveratrol—and piceid (3,4′,5-*trans*-trihydroxystilbene-3-O-β-mono-d-glucoside, RG)—the glucoside form of resveratrol. All compounds were purchased from Sigma-Aldrich Corp. or Cayman Chemical. All stilbenes were dissolved in ethanol, and the working concentrations of the tested compounds were obtained by adding a specified volume of concentrated solution to the culture medium.

### 4.3. Cytotoxicity Assays Using the MTT Test

The effect of resveratrol and its derivatives, piceatannol and piceid, on the proliferation of MCF-7 was estimated by the ability of the mitochondrial dehydrogenase of metabolically viable cells to reduce the tetrazolium salt 3-(4,5-dimethylthiazol-2-yl)-2,5-diphenyltetrazolium bromide (MTT) to form the blue formazan product. For this purpose, cells were seeded on 96-well plates (5000 or 10,000 cells per well) and cultured for 12–24 h. After 24 h, resveratrol, piceatannol, or piceid were added at appropriate concentrations (0–100 µM) and the incubation continued for 48 h.

For the combination of resveratrol or its derivative and IR treatment, cells were seeded into 40-mm tissue culture dishes (50,000 or 100,000 cells per dish) and cultured for 12–24 h. After this time, cells were pretreated with the indicated stilbene (at a concentration of 5 or 25 μM) for 3 h prior to exposure to IR. Then, the cells were incubated for 48 h. In our study, we used the following doses of IR: 2 Gy and 6 Gy. These are the standard clinical X-ray doses used in radiotherapy.

Afterward, 20 µL of MTT solution (5 mg/mL) was added to each well, or 200 µL of MTT solution was added per dish, and the plate or dish was incubated for 2 h. After this time the medium was removed, and the formazan crystals that had formed were dissolved with 100 µL of DMSO per well (or 1000 µL per dish). The absorbance of the plate was read at 570 nm, with a reference wavelength of 720 nm, using a microplate reader. The absorbance value was proportional to the number of viable cells in a sample. The absorbance of the control cells was assumed to be 100%.

### 4.4. Detection of Apoptosis and Necrosis by Flow Cytometry

Apoptotic, necrotic, and living cells were quantified by double staining with the Annexin V Apoptosis Detection Kit II, employing FITC-labelled Annexin V. Annexin V binds to cells that expose phosphatidylserine at their surface, a feature of cells that are undergoing apoptosis. Cells were seeded into 40-mm tissue culture dishes (500,000 cells per dish) and cultured for 12–24 h. The cells were preincubated with resveratrol or its derivative (at a concentration of 25 μM) for 3 h at 37 °C and then exposed to IR (6 Gy). Cells were incubated for 24 h. Then the cells were trypsinised and suspended in DMEM. After incubation of the cells with the compounds, the cells were washed twice with cold PBS and resuspended in 1 × binding buffer, and 5 µL of FITC-Annexin and 5 µL of propidium iodide were added. Later, the cells were vortexed gently and incubated (15 min) in the dark, and their fluorescence was measured within 1 h. All fluorescence measurements were done in a Becton Dickinson LSR II cytometer. Cells emitting weak green (FITC-Annexin) and weak red fluorescence (propidium iodide) were counted as apoptotic.

### 4.5. Antioxidant Enzyme Activity Assay

#### Preparation of Cell Lysates

For determination of the antioxidant enzyme activity, the cells were seeded into 40-mm tissue culture dishes at a density of 500,000 cells per dish and cultured for 12–24 h. The cells were preincubated with resveratrol and its derivatives (5 or 25 μM) for 3 h at 37 °C and exposed to IR (2 or 6 Gy). The cells were incubated for 48 h. Next, the cells were harvested and washed twice with cold PBS. The supernatant was discarded, and the cells were resuspended in 100 µL of protease inhibitor cocktail, which contained six broad-spectrum protease inhibitors: AEBSF, aprotinin, bestatin, E-64, leupeptin, and pepstatin A (Thermo Scientific 100 × Halt Protease Inhibitor Cocktail). The samples were frozen at −20 °C.

The CAT activity was assayed by monitoring the rate of disappearance of H_2_O_2_ at 240 nm [75]. One unit of CAT is the activity of catalase that catalyses the conversion of 1 µmol of hydrogen peroxide per minute.

The GPx activity was determined spectrophotometrically at 340 nm by measuring the rate of glutathione (GSH) oxidation by t-butyl hydroperoxide (tBOOH), according to method by Rice-Evans et al. (1991) [76].

The SOD activity was based on the ability of SOD to inhibit the autoxidation of epinephrine at alkaline pH. The oxidation of epinephrine was followed in terms of the production of adenochrome, which exhibits an absorption maximum at 480 nm [77]. One unit of SOD is described as the amount of enzyme required to cause 50% inhibition of epinephrine autoxidation per 300 μL of assay mixture.

All enzyme activities (CAT, GPx, and SOD) were expressed as specific activities (U/mg protein). The concentration of protein was measured according to the Bradford method. The absorbances of the samples were measured at 595 nm, and bovine serum albumin was used as a standard.

### 4.6. Gene Expression Analysis by Real-Time PCR

Total RNA was extracted by the Extractme Total RNA kit according to the manufacturer’s instructions. The concentration of extracted RNA was assayed using a NanoDrop spectrophotometer. All extracted samples were stored at −80 °C for further experiments. About 1 μg of extracted RNA was reverse transcribed into cDNA with a cDNA synthesis kit (RevertAid First Strand cDNA Synthesis Kit), according to the manufacturer’s instructions. The cDNA sample was kept at −20 °C until use.

The gene expressions of p53, Bcl-2, Bax, caspase 3, and caspase 8 were evaluated using the real-time PCR-based Eva Green assay. The HPRT gene was used as a housekeeping gene. Table 2 shows the sequences of primers used in the RT-PCR analysis. Real-time analysis was carried out using Eco 48 Real time PCR machine. Two microlitres of EvaGreen Supermix, 1 μL of cDNA, and 0.2 μL of each primer set were used for amplification in a 10 μL reaction mixture. All samples were amplified in triplicates. The cycling conditions were as follows: 12 s at 95 °C and 40 cycles at 95 °C for 15 s and 60 °C for 20 s.

### 4.7. Cell Lysate and Immunoblotting

In order to assess the expression of caspases 3, 8, and β-actin in MCF-7, after incubation with resveratrol, piceatannol and piceid cells were lysed (4 °C, 20 min) in a RIPA buffer containing 50 mmol/L Tris HCl pH 8, 150 mmol/L NaCl, 1% Nonidet P-40, 0.5% sodium deoxycholate, 0.1% SDS, 1 mmol/L EDTA, and 1 mmol/L PMSF (final concentration 10 μM). Protein concentration was determined using Lowry method [78]. After centrifugation, the supernatants were collected. Protein lysates (30 μg) were loaded to each lane, and the probes were electrophoretically separated by 8% and 12.5% sodium dodecyl sulfate-polyacrylamide gel electrophoresis (SDS-PAGE) and transferred to Immobilon P as described by Towbin et al. (1979). Subsequently, the membranes were blocked in 5% non-fat dry milk in TBST buffer (10 mM Tris-HCl, pH 7.5, 150 mM NaCl, and 0.05% Tween 20) for 1 h at room temperature. After blocking, the membranes were incubated overnight with antibodies specific to caspases 3 and 8 (1:500 dilution; Santa Cruz Biotechnology) and β-actin (1:1000 dilution; Santa Cruz Biotechnology) in TBST buffer in a cold room. After incubation with the primary antibody, the membranes were washed with TBST and incubated with appropriate secondary antibodies (1:5000 dilution; Sigma Aldrich) conjugated with alkaline phosphatase in TBST for 2 h at room temperature. After incubation, the membranes were then washed several times with TBST, and the proteins were visualised by incubation with the substrate solution (0.33 mg/mL of nitroblue tetrazolium, 0.17 mg/mL of 5-bromo-4-chloro-3-indolyl phosphate in 100 mM Tris-HCl, pH 9.5, 100 mM NaCl, and 5 mM MgCl_2_).

## Figures and Tables

**Table 1:** Effect of resveratrol (R), piceatannol (ROH), and piceid (RG) at concentrations of 25 μM, IR (dose of 6 Gy), and their combinations on the transcription of genes involved in the apoptosis of MCF-7 cells. Gene expression was normalised to the HPRT housekeeping gene. The delta–delta Ct method was used to determine the relative levels of mRNA expression between the experimental samples and controls. The mean difference (*) was compared with the control and (#) with IR.

	Control	6 Gy	R	R + 6 Gy	ROH	ROH + 6 Gy	RG	RG + 6 Gy
***p53***	1.00 ± 0.13	1.56 ± 0.11 *	1.78 ± 0.28 *	1.97 ± 0.20 *#	1.64 ± 0.27 *	1.74 ± 0.15 *	1.27 ± 0.12 *	1.50 ± 0.19 *
***Caspase 3***	1.00 ± 0.05	1.24 ± 0.12 *	1.19 ± 0.30	1.25 ± 0.16	1.04 ± 0.16	1.28 ± 0.03 *	0.87 ± 0.10	1.26 ± 0.16
***Caspase 8***	1.00 ± 0.04	1.50 ± 0.31 *	1.41 ± 0.42 *	2.07 ± 0.11 *#	1.13 ± 0.44	2.12 ± 0.12 *#	1.73 ± 0.41 *	1.94 ± 0.03 *#
***Bax***	1.00 ± 0.03	2.73 ± 0.26 *	2.36 ± 0.27 *	5.25 ± 0.46 *#	2.17 ± 0.46 *	4.05 ± 0.53 *#	1.48 ± 0.11 *	3.19 ± 0.42 *#
***Bcl-2***	1.00 ± 0.05	1.63 ± 0.26 *	1.53 ± 0.31 *	2.18 ± 0.39 *#	1.52 ± 0.31 *	2.02 ± 0.36 *#	1.25 ± 0.28 *	1.65 ± 0.26 *

**Table 2:** The primer sequences of genes.

Primer	Sense	Antisense
***HPRT***	ATGGACAGGACTGAACGTCTT	TCCAGCAGGTCAGCAAAGAA
***p53***	TAACAGTTCCTGCATGGGCGGC	AGGACAGGCACAAACACGCACC
***Bcl-2***	TTGTGGCCTTCTTTGAGTTCGGTG	GGTGCCGGTTCAGGTACTCAGTCA
***Bax***	CCTGTGCACCAAGGTGCCGGAACT	CCACCCTGGTCTTGGATCCAGCCC
***Caspase 3***	TGGACTGTGGCATTGAGAC	CAAAGCGACTGGATGAACC
***Caspase 8***	CTGGATGATGACATGAACCTGCTG	GCTCTTGTTGATTTGGGCACAGAC

## Data Availability

All the data available is in the manuscript.
